# *Notes from the Field:* High Volume of Lyme Disease Laboratory Reporting in a Low-Incidence State — Arkansas, 2015–2016

**DOI:** 10.15585/mmwr.mm6642a8

**Published:** 2017-10-27

**Authors:** Natalie A. Kwit, Elizabeth A. Dietrich, Christina Nelson, Richard Taffner, Jeannine Petersen, Martin Schriefer, Paul Mead, Susan Weinstein, Dirk Haselow

**Affiliations:** ^1^Epidemic Intelligence Service, CDC; ^2^Division of Vector-Borne Diseases, National Center for Emerging and Zoonotic Infectious Diseases, CDC; ^3^Laboratory Leadership Service, CDC; ^4^Arkansas Department of Health.

Although Arkansas lies within the geographic range of the principal Lyme disease tick vector, *Ixodes scapularis*, because of ecologic and entomologic factors, the risk for human infection is low, and no confirmed Lyme disease cases were reported in Arkansas during 2008–2014 (*1*). However, during 2015–2016, the Arkansas Department of Health (ADH) received several hundred potentially positive serologic laboratory reports for Lyme disease. Recommended serologic testing for Lyme disease is a two-tiered process; only if the first-tier enzyme immunoassay is positive or equivocal should the second-tier western blot be performed. A positive overall result can only be concluded when results of both individual tests are documented (*2*). Laboratory reports submitted to ADH during 2015–2016 did not always include complete or overall positive two-tiered serology results or associated clinical information needed to make a case determination. To facilitate Lyme disease surveillance in the setting of a high volume of reports and to ascertain whether local transmission of Lyme disease has occurred, ADH and CDC reviewed laboratory reports and clinical data, classified cases according to the surveillance definition, and investigated cases with potential for confirmation of Lyme disease.

Paper laboratory reports of Lyme disease testing sent to ADH were matched by patient name and birth date with electronic laboratory surveillance data to consolidate reports. Reports were then sorted and prioritized for follow-up based on recommended laboratory criteria for diagnosis and available information. Among the 911 Lyme disease laboratory reports submitted to ADH during 2015–2016, a total of 582 combined reports for unique patients were identified. Among 295 reports with sufficient information to make a determination, 282 (95.6%) did not meet the Council of State and Territorial Epidemiologists surveillance criteria for Lyme disease.* Eleven (3.7%) met the probable (three reports) or suspected (eight) Lyme disease surveillance case definition, and two reports (0.7%) met the confirmed case definition. Further investigation of the two confirmed cases revealed that both patients were likely infected in high-incidence states. One patient had signs of arthritis soon after moving to Arkansas from the northeastern United States, but did not receive a diagnosis of and treatment for Lyme disease until nearly 1 year later, underscoring the fact that even where Lyme disease is rare, providers need to obtain a travel history and consider the diagnosis in patients with compatible symptoms who have lived in or visited states where Lyme disease is common.

Lyme disease is the most common vectorborne disease in the United States, caused by the spirochete *Borrelia burgdorferi* sensu stricto and the recently discovered *Borrelia mayonii* (*3*), but risk for infection is not uniform. In 2015, 95% of cases in the United States were reported from 14 states concentrated in the Northeast, mid-Atlantic, and upper Midwest regions (*1*). In Arkansas, host-seeking *I. scapularis* ticks are much less abundant, less prone to biting humans, rarely infected with *B. burgdorferi*, and prefer feeding on nonreservoir hosts (*4*). However, the occurrence of travel-related infections and the need to monitor for emergence of locally acquired infection underscore the importance of Lyme disease surveillance in Arkansas and other low-risk states.

Of the hundreds of Lyme disease reports submitted to ADH during 2015–2016, many had incomplete information or negative laboratory results; however, the ADH Lyme disease surveillance system did identify two confirmed, travel-associated infections. The absence of similarly confirmed, locally acquired cases supports the view that autochthonous transmission of Lyme disease is either exceedingly rare or has not occurred in Arkansas. The risk for other tickborne diseases in Arkansas results in frequent requests for Lyme disease testing as part of a general tickborne disease serologic panel, even when Lyme disease is not suspected by the clinician. Strong clinical evidence supported by positive two-tiered serologic testing is essential to securing a diagnosis of Lyme disease in low-incidence states (*2**,**5*).

For reporting Lyme disease to public health authorities, health care providers should follow infectious disease testing recommendations and reporting guidelines set forth by state health departments and only submit reports for cases that have complete and positive test results and associated clinical information. Given that multiple laboratory tests, potentially performed and reported by different laboratories, might be necessary to determine Lyme disease case status, health departments need an efficient process to manage and interpret incoming laboratory reports.
